# Preventive Effect of *Anemarrhenae rhizome* and *Phellodendri cortex* on Danazol-Induced in Precocious Puberty in Female Rats and Network Pharmacological Analysis of Active Compounds

**DOI:** 10.3390/plants11010023

**Published:** 2021-12-22

**Authors:** Kyeong Ri Kim, Tuy An Trinh, Ji Yun Baek, Dahae Lee, Sehun Lim, Jonghyup Kim, Won-Yung Lee, Chang-Eop Kim, Ki Sung Kang, Hye Lim Lee

**Affiliations:** 1 Department of Pediatrics, College of Korean Medicine, Daejeon University, Daejeon 300716, Korea; krkimcmm@naver.com; 2 Saigon Pharmaceutical Science and Technology Center, University of Medicine and Pharmacy at Ho Chi Minh City, Ho Chi Minh 70000, Vietnam; ttan@ump.edu.vn; 3 Department of Food Science, Gyeongnam National University of Science and Technology, Jinju 52725, Korea; wldbsttn@naver.com; 4 College of Korean Medicine, Gachon University, Seongnam 13120, Korea; pjsldh@naver.com (D.L.); lwy21@gachon.ac.kr (W.-Y.L.); eopchang@gachon.ac.kr (C.-E.K.); 5 Department of Anesthesiology and Pain Medicine, College of Medicine, Inje University, Busan 50834, Korea; anespc@naver.com (S.L.); pet93@naver.com (J.K.)

**Keywords:** *Anemarrhenae rhizome*, *Phellodendri cortex*, precocious puberty, danazol, network pharmacology

## Abstract

*Anemarrhenae rhizome* and *Phellodendri cortex* have historically been used for the treatment of precocious puberty (PP) in oriental medicine. Our study aimed to evaluate the effect of APE, a mixture of the extracts from these herbs, against danazol-induced PP in female rats. The offspring were injected danazol to establish the PP model, and then treated with APE daily, and observed for vaginal opening. At the end of the study, the levels of gonadotropic hormones, such as estradiol, follicle-stimulating hormone, and luteinizing hormone, were determined by ELISA. Moreover, the mRNA expression of GnRH, netrin-1, and UNC5C in hypothalamic tissues was determined by real-time PCR. Network pharmacological analysis was performed to predict the active compounds of APE and their potential actions. APE treatment delayed vaginal opening in rats with PP. In addition, APE treatment reduced LH levels and suppressed UNC5C expression. Gene set enrichment analysis revealed that the targets of APE were significantly associated with GnRH signaling and ovarian steroidogenesis pathways. In conclusion, APE may be used as a therapeutic remedy to inhibit the activation of the hypothalamic–pituitary–gonadal axis.

## 1. Introduction

Precocious puberty is an endocrine disorder characterized by the onset of secondary sexual characteristics before the age of eight years in girls and nine years in boys [1]. The incidence of precocious puberty in girls is higher than that in boys [2]. Precocious puberty is classified into three major types: central precocious puberty (CPP) that is gonadotropin-dependent, peripheral precocious puberty that is gonadotropin-independent, and normal variant puberty [3]. The early activation of the hypothalamic–pituitary–gonadal axis (HPGA) leads to the release of gonadotropins that initiate the development of secondary sexual characteristics and accelerate bone maturation in individuals with CPP [4]. Therefore, CPP can be distinguished from the other two types of precocious puberty by hormone test and bone age determination [1].

The incidence and prevalence of CPP in Korea were investigated based on the national registry data from the Health Insurance Review and Assessment Service. Between 2008 and 2014, 37,890 girls and 1220 boys were diagnosed with CPP nationwide. The incidence rate (per 100,000 children) of CPP during this period was 262.8 for girls and 7.0 for boys. The overall prevalence of CPP during the period 2008–2014 was 410.6 for girls and 10.9 for boys. Moreover, this epidemiologic study showed that the annual incidence of CPP among Korean children rapidly increased by the year during 2008–2014 in both girls (from 89.4 to 415.3) and boys (from 1.6 to 14.7) [5].

Until now, gonadotrophin-releasing hormone analog (GnRHa) is the only effective treatment for CPP among the currently available, which maintains the stable level of GnRH to reduce the steroid hormones to the prepubertal level. Several synthetic peptide drugs, such as leuprolide, triptorelin, and goserelin, have been used clinically [6,7]. GnRHa continuously stimulates the anterior pituitary through GnRH receptors. The long-term continuous stimulation of the anterior pituitary downregulates its responsiveness, which suppresses the production of luteinizing hormone (LH) and follicle-stimulating hormone (FSH) [8,9]. In this way, administration of GnRHa reduces the secretion of gonadal sex steroids to the prepubertal levels in individuals with CPP. However, various common side effects of GnRHa have been reported, such as injection site reactions, sterile abscess, pain, bruising, and nausea [10]. In addition, there are no clear evidence of the effect of GnRHa treatment on increasing the adult height of girls with CPP aged between 6 and 8 years [7,8].

In Chinese medicine, some medicinal plants have been used as an alternative therapy for the treatment of CPP, including *Anemarrhenae asphodeloides* rhizome (Ji-Mo) and *Phellodendri cortex* (Huang Bai) [11,12,13,14,15]. The combination treatment with the Western and Oriental medicines may be an effective strategy for CPP treatment. Many research and clinical trials have been conducted to evaluate the effects of herbal medicines on CPP [15,16,17]. In this study, we investigated the effect of the extract of *Anemarrhenae asphodeloides* rhizome and *Phellodendri cortex*, abbreviated as APE, against CPP in an animal model represented by delayed vaginal opening and reduced release of gonadotropic hormones against danazol-induced precocious puberty in female rats. Simultaneously, network pharmacological analysis was used to explore the active compounds of APE and their potential in alleviating precocious puberty symptoms.

## 2. Results

### 2.1. Cytotoxicity Test of APE on GT1-7 Cells

**Figure 1:**
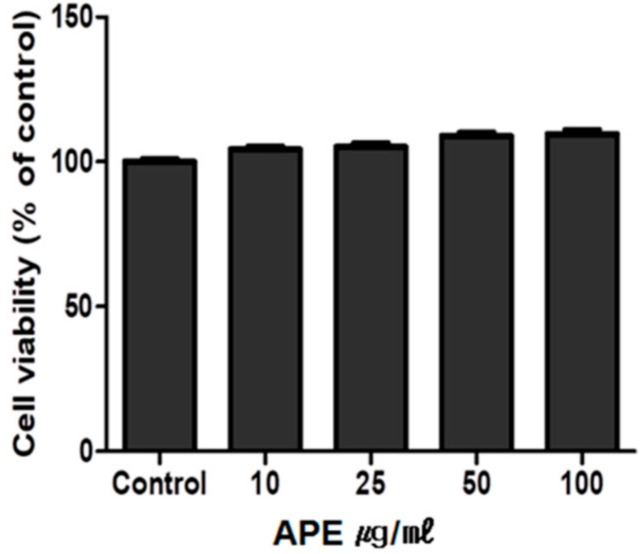
Cytotoxicity test of APE on GT1-7 cells. GT1-7 cells were seeded in a 96-well plate and then treated with APE at 10–100 μg/mL to evaluate its cytotoxic effect.

GT1-7 is an immortalized mature mouse hypothalamic GnRH neuronal cell line. GT1-7 cells can be used as an in vitro model of GnRH-secreting neurons in the hypothalamus [18]. The cytotoxic effect of APE on GT1-7 cells was evaluated using cell viability assay. As shown in Figure 1, cell viability was not significantly affected after treatment with 10–100 μg/mL APE. Thus, APE up to a concentration of 100 μg/mL was not cytotoxic to GT1-7 cells.

### 2.2. Effect of APE on Vaginal Opening

**Figure 2:**
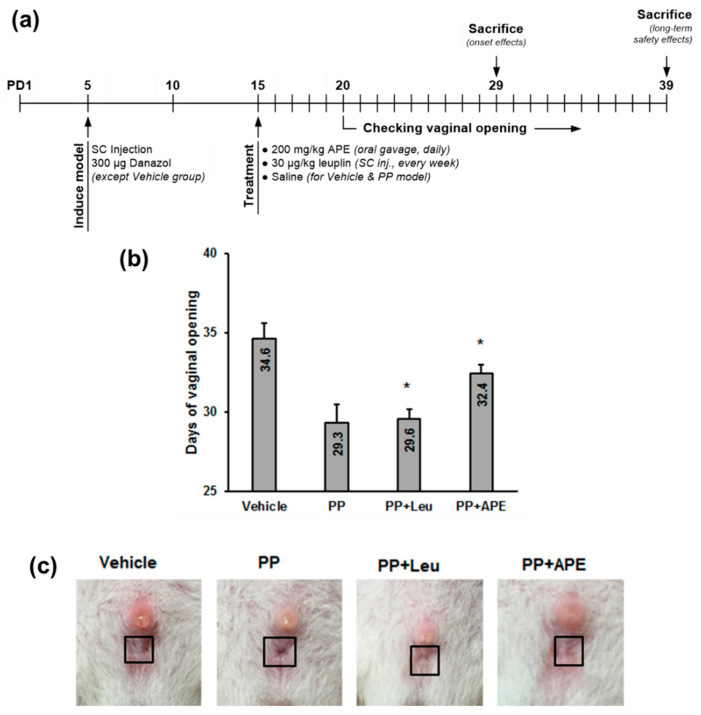
APE delayed vaginal opening in danazol-treated female rats. (**a**) Schematic representation of the experimental design. (**b**) Treatment with the APE extract delayed vaginal opening. (**c**) The representative images of vagina of rats in different experimental groups on postnatal day 29. * *p* < 0.05, compared to the PP group.

Female laboratory rats were injected danazol on PD 5 to establish the PP model. To precisely define the onset of puberty, vaginal opening was chosen as a reliable sign of sexual maturation. Vaginal opening was observed in danazol-induced PP rats on PD 29, five days earlier than in the vehicle group (Figure 2). Vaginal opening was significantly delayed in the APE-treated group to PD 32. Thus, APE showed an inhibitory effect on PP in female rats.

### 2.3. Body Weight, Body Length, and ALP Level

**Figure 3:**
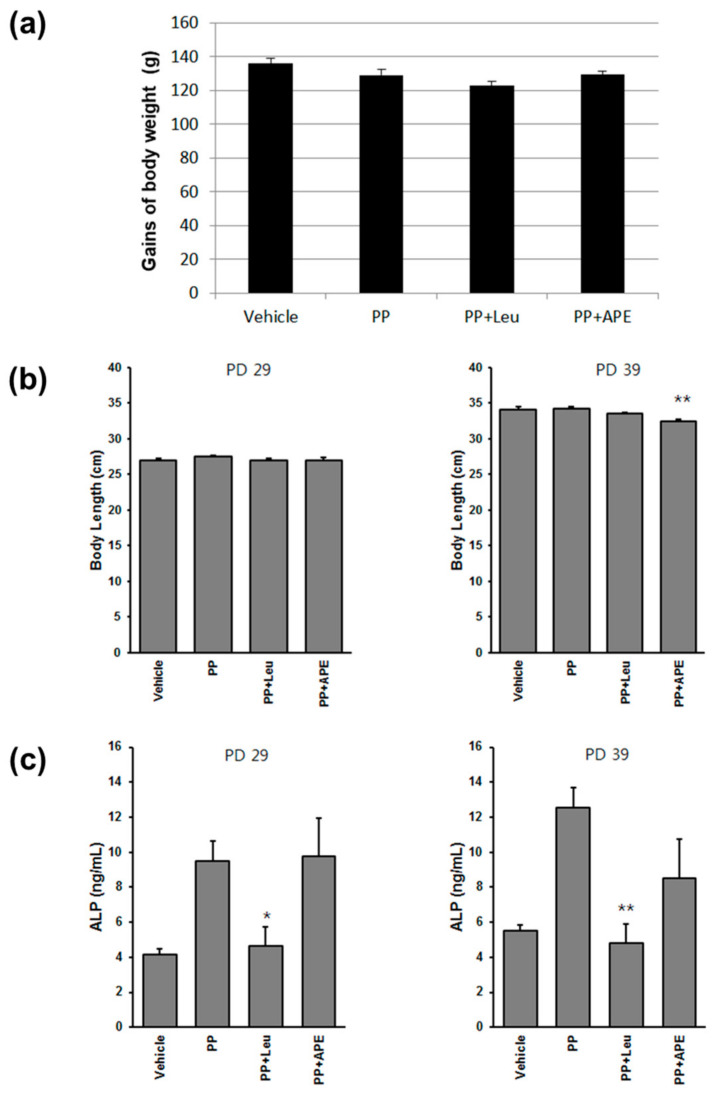
Effect of APE on the growth rate of experimental rats. (**a**) The body weight gain in rats of different groups on PD 39. (**b**) Effect of APE on body length of rats in the PP model. (**c**) The serum ALP levels in rats of different groups. * *p* < 0.05; ** *p* < 0.01, compared to the PP group.

There was no significant difference in body weight gain among different experimental groups (Figure 3a). Accelerated growth, which results in a restricted final stature, is an important sign of PP. The results revealed that, compared with the other groups, treatment with APE in the long term slightly reduced the body length of rats (Figure 3b). We also measured the serum ALP level, a marker of bone maturation [19], to evaluate the effect of APE on regulating the growth rate. Only leuplin showed an inhibitory effect to restore the serum ALP concentration to the basal level; however, APE treatment did not change the serum ALP level in the PP model (Figure 3c).

### 2.4. Organ Index of Uterus, Pituitary, and Hypothalamus

**Figure 4:**
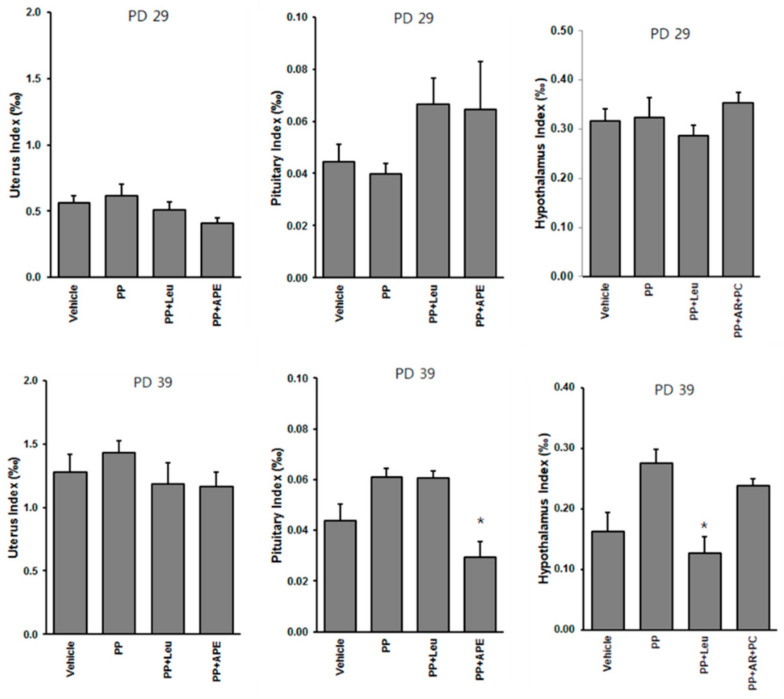
Effect of APE on the organ index of uterus, pituitary, and hypothalamus of experimental rats. * *p* < 0.05, compared to the PP group.

The organ index of the uterus did not vary significantly among different experimental groups. The pituitary index was decreased after treatment with APE for the long term, as shown in Figure 4. In addition, treatment with leuplin reduced the hypothalamus index compared with that of the PP group. Leuplin is a sustained-release injectable formulation of leuprorelin acetate that is commonly used for the treatment of hormone-dependent diseases such as prostate cancer, premenopausal breast cancer, transgender hormone therapy, and early puberty [8].

### 2.5. Effect of APE on Serum Hormone Levels in Rats

**Figure 5:**
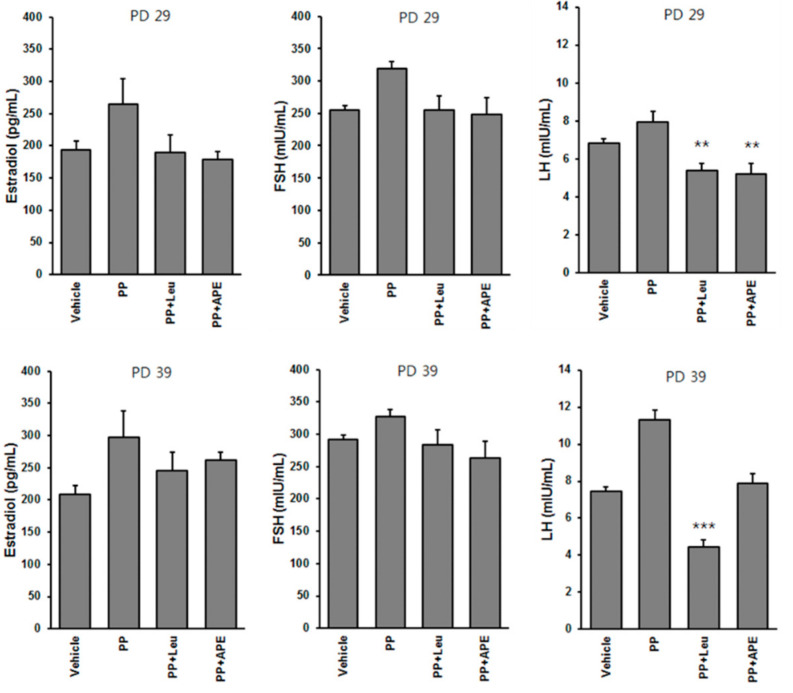
Effect of APE on serum gonadotropic hormone levels in experimental rats. ** *p* < 0.01; *** *p* < 0.001, compared to the PP group.

The onset of PP is marked by the activation of the HPGA, which leads to an increase in gonadotropic hormones, such as E2, FSH, and LH. There was no significant change in the serum E2 level between the APE-treated group and the non-treated group. Neither leuplin or APE affected the level of FSH in rats with PP. The results revealed that treatment with APE only suppressed LH secretion in the early stages of PP (Figure 5).

### 2.6. Effect of EIF on Hypothalamic GnRH, Netrin-1, and UNC5C mRNA Expressions

**Figure 6:**
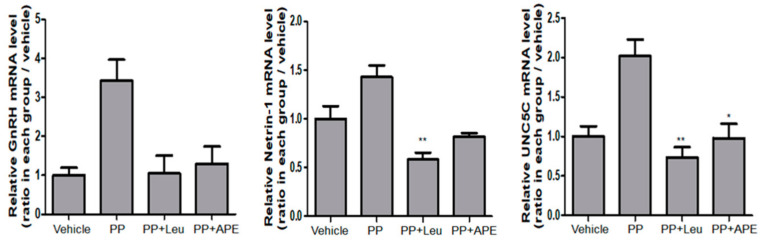
Effect of APE on mRNA expression of hypothalamic GnRH, netrin-1, and UNC5C. * *p* < 0.05; ** *p* < 0.01, compared to the PP group.

The hypothalamic tissues were collected from the brains of experimental rats on PD 29 to evaluate the mRNA expression of GnRH, netrin-1, and UNC5C. In the hypothalamus of rats with PP, the expression levels of netrin-1 and its receptor UNC5C were enhanced, which resulted in the release of GnRH [20]. As shown in Figure 6, treatment with APE reduced the expression of GnRH, netrin-1, and UNC5C. The mRNA level of UNC5C was notably decreased after APE treatment.

### 2.7. Identifying Bioactive Compounds and Targets of APE

We employed TCMSP to construct the herb–compound–target network [21]. A total of 54 compounds was identified from *Anemarrhenae rhizome* and *Phellodendri cortex*. Oral bioavailability and drug-likeness thresholds (≥30% and 0.18%, respectively) were applied to screen the compounds having potential in vivo medicinal effects. We found 15 compounds that met the threshold and identified 104 relevant targets for the selected compounds. The list of compounds and their data are shown in Table 1.

### 2.8. Pathway Enrichment Analysis of APE

**Figure 7:**
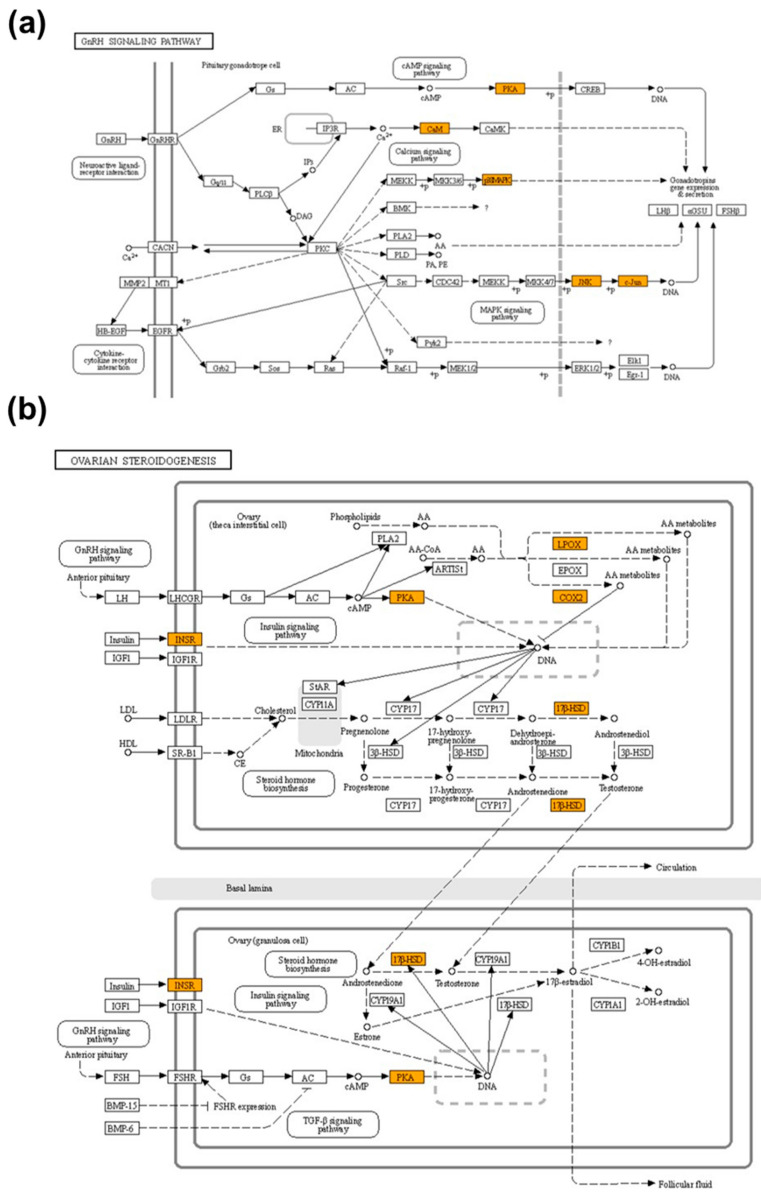
The target genes of APE active compounds in the GnRH signaling and ovarian steroidogenesis pathways. (**a**) The GnRH signaling pathway. (**b**) The ovarian steroidogenesis pathway in theca-interstitial cells and granulosa cells. The orange-colored box represents a predicted target gene of APE active compounds.

Gene set enrichment analysis (GSEA) was performed on the KEGG database to identify potential pathways of the active compounds from *Anemarrhenae rhizome* and *Phellodendri cortex* [22,23]. The GnRH signaling and ovarian steroidogenesis pathways were chosen to predict the protein targets related to the therapeutic effect of APE on PP. We found that the numbers of related targets for GnRH signaling and ovarian steroidogenesis pathways were five each, which was significantly associated with both pathways (Table 2). The targets of APE active compounds in GnRH signaling and ovarian steroidogenesis pathways were visualized using KEGG Mapper (Figure 7).

### 2.9. Herb–Compound-Target Network of APE

**Figure 8:**
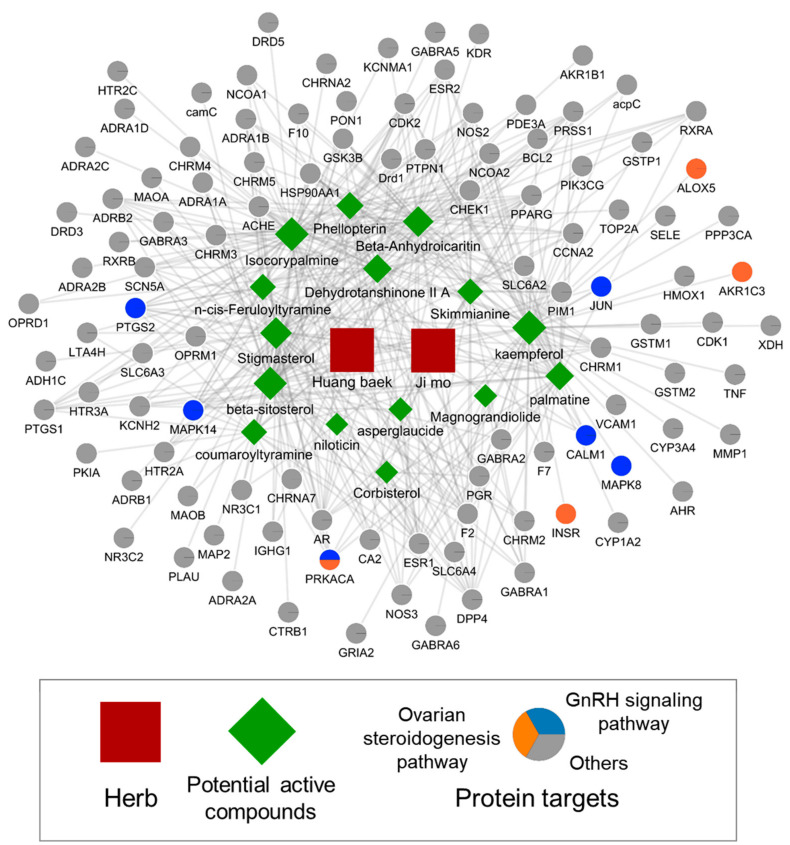
Compound–target network of APE. The targets genes of the GnRH signaling and ovarian steroidogenesis pathways are presented by colored nodes. Genes directly associated with PP are bordered in the network. The size of each node is proportional to its interaction potential.

We constructed and visualized herb–compound–target network of APE using Cytoscape [24], as shown in Figure 8. The network consisted of 121 nodes and 412 edges, in which nodes denote herbs or active compounds or protein targets (2, 15, and 104, respectively), and edges denote a herb–compound or compound–target relationship (16 and 396, respectively). Among the targets related to GnRH signaling and ovarian steroidogenesis pathway, the protein targets showing the highest degree were PTGS2, MAPK14, PRKACA, and CALM1 (11, 9, 7, and 5, respectively). Those targets are expected to be cumulatively affected by the compounds of APE. Among the active compounds of APE, kaempferol, β-sitosterol, palmatine, and isocorypalmine were found to be closely connected to targets involved in the GnRH signaling and ovarian steroidogenesis pathway (8, 4, 4, and 4, respectively, as a number of compound–target interactions). These compounds are predicted to be active compounds that mainly contribute to the effect of APE on PP.

## 3. Discussion

The results of in vivo study indicated that APE treatment delayed vaginal opening in rats with PP by inhibiting the activation of HPGA. APE inhibited the increase in the pituitary index and LH serum level at the onset of PP. Moreover, APE reduced mRNA expression of UNC5C in hypothalamic tissues, which is involved in the regulation of GnRH release. According to the previous references on the TCMPS database, 15 active compounds from APE were selected as subjects for GSEA to predict their target genes associated with the GnRH signaling and ovarian steroidogenesis pathways. Herbal remedies are more popular than acupuncture and manipulative therapies for adjuvant treatment of CPP in children. Zhi-Bai-Di-Huang-Wan is the most commonly prescribed formulation, accounting for 23.73% of medical indications [25]. Zhi-Bai-Di-Huang-Wan consists of eight herbs, including *Anemarrhenae rhizome* and *Phellodendri cortex*. Our study also proved that the extract of these medicinal plants had pharmacological effects in female rats with PP.

The PP rat model has been used in several previous studies to evaluate the effects of herbal mixtures from Oriental medicine. Recently, Bai et al. studied the effect of the Fuyou formula, containing *Anemarrhenae rhizome* and *Rehmanniae radix*, on GT1-7 cells and danazol-induced PP rats [26]. The results showed that the Fuyou formula alleviated the symptoms in rats with PP and downregulated the GPR54/GnRH signaling pathway in GT1-7 cells. In another study, Yin et al. evaluated the therapeutic effect of the Shugan Xiehuo formula containing 10 herbs in a female rat model with PP [27]. Treatment with Shugan Xiehuo reduced the serum level of gonadotropic hormones in PP rats and suppressed the expression of GnRH, GnRH receptor, estrogen receptor subtype α, and G protein-coupled receptor 30 in the hypophysis. Our study, in line with previous studies, showed the effectiveness of herbal remedies for the treatment of PP via inhibition of the HPGA activation using the same animal model.

APE may be a promising candidate for the supportive treatment of PP. However, additional preclinical trials need to be conducted to evaluate the safe clinical dose, pharmacokinetics, and bioavailability of this extract. According to the network pharmacological analysis results, the mechanism of action of APE against PP should be confirmed by cell experiments or in vivo studies. Moreover, the formula of APE needs to be optimized and developed based on the results of preclinical trials.

## 4. Materials and Methods

### 4.1. Plant Materials

The *Anemarrhenae rhizome* and *Phellodendri cortex* were purchased from Omniherb Co., Ltd. (Daegu, Korea). Dried medicinal plant powder (500 g) was extracted three times with 80% methanol (1.5 L) at 25 °C and then dried under reduced pressure to obtain the freeze-dried extract. The extracts of *Anemarrhenae rhizome* and *Phellodendri cortex* were mixed in a 1:1 ratio for use in cell and animal experiments.

### 4.2. Cytotoxicity Test

The hypothalamic cells GT1-7 (Burlington, MA, USA) were grown in Dulbecco’s modified Eagle’s medium (Corning, Manassas, VA, USA), supplemented with 10% fetal bovine serum (Atlas Biologicals, Fort Collins, CO, USA), 100 U/mL of penicillin, and 100 mg/mL of streptomycin (Gibco, Grand Island, NY, USA). Cells were maintained at 37 °C in a humidified atmosphere under 5% CO_2_ and sub-cultured every 2 days. GT1-7 cells were seeded in a 96-well plate and then treated with APE at concentrations ranging from to 10–100 μg/mL to evaluate the cytotoxic effect. After 24 h of treatment, cell viability was determined by adding 10 μL of EZ-Cytox assay reagent (DoGen, Seoul, Korea) to each well, and the absorbance was measured at 450 nm using a microplate reader (PowerWave XS; Bio-Tek Instruments, Winooski, VT, USA) [28].

### 4.3. Animal Experimental Design

Our animal experimental protocol was in accordance with the Guidelines for the Care and Use of Laboratory Animals of the National Institutes of Health. This protocol was approved by the Institutional Animal Care and Use Committee of Gachon University (GIACUC-R2019035). For the experiment, 2-day-old Sprague-Dawley female offspring rats with their mothers were purchased from Daehan Biolink (Chungcheongbuk-do, Korea) and housed under a 12 h–12 h light–dark cycle at 20–22 °C. The rats were randomly divided into four different groups: vehicle, precocious puberty (PP) model, PP model treated with leuprorelin acetate (Leuplin; reference drug), and PP model treated with APE. There were 6 animals in each group. On the 5th postnatal day (PD), the offspring were subcutaneously injected 300 μg of danazol (Sigma-Aldrich, St. Louis, MO, USA), which was dissolved in 30 μL of vehicle (propylene glycol: ethanol, 1:1, v/v) to establish the PP model, and the vehicle group was injected solvent only. The test group was orally administered APE at a dose of 200 mg/kg, whereas the positive control group was injected with Leuplin at a dose of 30 μg/kg from PD 15 onwards. The vaginal opening of all rats was observed daily.

### 4.4. Blood Sample, Organ, and Brain Tissue Collection

The rats were sacrificed on PD 29 or 39 depending on the purpose of the experiments. Blood samples were collected from the abdominal aorta to test the gonadal hormone levels in the serum. The uterus, pituitary gland, and hypothalamus tissues were harvested and weighed. The organ index was calculated as the ratio of organ weight to whole body weight. The samples were stored at −80 °C until further analysis.

### 4.5. Quantification of Serum Hormone Levels

The blood sample was centrifuged at 3000 rpm for 15 min at 4 °C to collect the serum. The levels of estradiol (E2), LH, FSH, and alkaline phosphatase (ALP) were measured using ELISA kits (Abebio, Wuhan, China), according to the manufacturer’s instructions [29]. The standard curve of each hormone was prepared using the Curve Exert 1.4 software. The concentrations of serum hormones were determined using the linear method.

### 4.6. Real-Time Polymerase Chain Reaction

The hypothalamic tissues were homogenized to extract the total RNA using TRIzol reagent (Gibco, Waltham, MA, USA). An equal amount of the total RNA was used to synthesize cDNA using the RevertAid First Strand cDNA Synthesis Kit (Thermo Scientific, Waltham, MA, USA) [30,31]. The reaction mixture for real-time PCR consisted of 1 μL of cDNA, 10 μL of PowerUp^TM^ SYBR Green Master Mix (Thermo Scientific, Waltham, MA, USA), and a set of primers at a final concentration of 200 nM. The specific primer sequences used are listed in Table 3. Real-time PCR was performed on QuantStudio^TM^ Real-Time PCR System (Applied Biosystems, Waltham, MA, USA) with the standard cycling mode as follows: 50 °C for 2 min (UDG activation); 95 °C for 2 min (dual-lock DNA polymerase activation); and 40 cycles with 3 steps included denaturation at 95 °C for 15 s, annealing at 60 °C for 15 s, and extension at 72 °C for 1 min. The relative expression levels of the target genes were calculated using the double Ct value method [20], and β-actin was used as a housekeeping gene.

### 4.7. Construction of Herb–Compound–Target Network

Information about the constituents of APE and its targets was obtained from the Traditional Chinese Medicine Systems Pharmacology (TCMSP) database [21]. Oral bioavailability and drug-likeness thresholds were applied to exclude compounds that were less likely to have in vivo medicinal effects. In TCMSP, the compound–target interactions include experimentally verified interactions and predicted results. The prediction was performed based on a validated machine-learning model (support vector machine and random forest).

### 4.8. Pathway Enrichment Analysis

The pathway of APE was analyzed using the Kyoto Encyclopedia of Genes and Genomes (KEGG) database. The Bonferroni correction was applied to correct the family wise error (FWE) generated in multiple tests according to the adjusted *p*-value.

### 4.9. Network Visualization

The interaction network between APE components and their target genes was visualized using the Cytoscape program. In the process of visualization, the targets involved in different pathways related to precocious puberty are displayed in different colors.

### 4.10. Statistical Analysis

Data are presented as the mean ± SD. Statistical analysis was conducted using GraphPad Prism (GraphPad, San Diego, CA, USA). The nonparametric Kruskal–Wallis test was used to compare the experimental groups, and Dunn’s post-hoc test was used to verify the statistical difference at the level of *p* < 0.05, *p* < 0.01, and *p* < 0.001.

## Figures and Tables

**Table 1:** List of compounds from *Anemarrhenae rhizome* and *Phellodendri cortex*.

Name of Compound	Pubchem ID	Structure	OB (%)	DL (%)
Niloticin	44559946	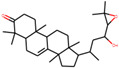	41.41427	0.81833
Stigmasterol	5280794	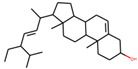	43.82985	0.75665
β-sitosterol	222284	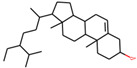	36.91391	0.75123
Corbisterol	12303924	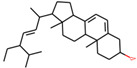	37.42312	0.75103
Palmatine	19009	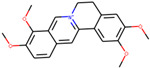	64.60111	0.64524
Isocorypalmine	440229	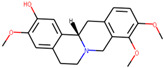	35.76844	0.59227
Asperglaucide	10026486	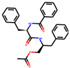	58.0163	0.51972
Beta-Anhydroicaritin	14583584	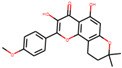	45.41193	0.43786
Dehydrotanshinone IIA	128994	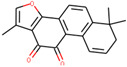	43.76229	0.40019
Phellopterin	98608	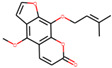	40.18556	0.27878
n-cis-feruloyltyramine	6440659	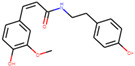	118.3477	0.26399
Kaempferol	5280863	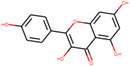	41.88225	0.24066
Coumaroyltyramine	13939145	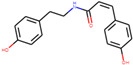	112.9016	0.20234
Skimmianine	6760	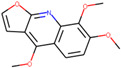	40.13655	0.19638
Magnograndiolide	5319198	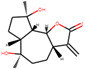	63.70888	0.18833

OB: oral bioavailability; DL: drug likeness.

**Table 2:** Significant enrichment pathways and their target genes related to the therapeutic effect of APE on PP.

Pathway	Overlap	*p*-Value	Adjusted *p*-Value	Targets (Gene Symbol)
GnRH signaling	5/93	0.00012	0.00059	MAPK8; JUN; PRKACA; MAPK14; CALM1
Ovarian steroidogenesis	5/49	5.49 × 10^−0.6^	4.45 × 10^−5^	ALOX5; INSR; AKR1C3; PRKACA; PTGS2

**Table 3:** List of primers used in real-time PCR.

Gene	Primer (5′-3′)
GnRH	F: GGCAAGGAGGAGGATCAAA R: CCAGTGCATTACATCTTCTTCTG
Netrin-1	F: AGAGTTTGTGGATCCGTTCG R: TTCTTGCACTTGCCCTTCTT
UNC5C	F: CACGACTCTCAGATACAGC R: TTCTTGGATTGGAGGACCAG
β-actin	F: CACCCGCGAGTACAACCTCC R: CCCATACCCACCATCACACC

(F: forward; R: reverse).

## Data Availability

Data is contained within the article.
